# Future of Nanotechnology in Food Industry: Challenges in Processing, Packaging, and Food Safety

**DOI:** 10.1002/gch2.202200209

**Published:** 2023-02-21

**Authors:** Rajesh Singh, Shradha Dutt, Priyanka Sharma, Ashok K. Sundramoorthy, Aman Dubey, Anoop Singh, Sandeep Arya

**Affiliations:** ^1^ Food Craft Institute Department of Skill Development Nagrota Jammu Jammu and Kashmir 181221 India; ^2^ School of Sciences Cluster University of Jammu Jammu Jammu and Kashmir 180001 India; ^3^ School of Hospitality and Tourism Management University of Jammu Jammu Jammu and Kashmir 180006 India; ^4^ Centre for Nano‐Biosensors Department of Prosthodontics Saveetha Dental College and Hospitals Saveetha Institute of Medical and Technical Sciences Chennai Tamil Nadu 600077 India; ^5^ Department of Physics University of Jammu Jammu Jammu and Kashmir 180006 India

**Keywords:** food safety, food security, hotel industry, nanomaterials, nanotechnology

## Abstract

Over the course of the last several decades, nanotechnology has garnered a growing amount of attention as a potentially valuable technology that has significantly impacted the food industry. Nanotechnology helps in enhancing the properties of materials and structures that are used in various fields such as agriculture, food, pharmacy, and so on. Applications of nanotechnology in the food market have included the encapsulation and distribution of materials to specific locations, the improvement of flavor, the introduction of antibacterial nanoparticles into food, the betterment of prolonged storage, the detection of pollutants, enhanced storage facilities, locating, identifying, as well as consumer awareness. Labeling food goods with nano barcodes helps ensure their security and may also be used to track their distribution. This review article presents a discussion about current advances in nanotechnology along with its applications in the field of food‐tech, food packaging, food security, enhancing life of food products, etc. A detailed description is provided about various synthesis routes of nanomaterials, that is, chemical, physical, and biological methods. Nanotechnology is a rapidly improving the field of food packaging and the future holds great opportunities for more enhancement via the development of new nanomaterials and nanosensors.

## Graphical abstract

This article describes the role of nanotechnology in the field of the food industry. Nanotechnology has greatly affected food packaging, food security, food processing, etc. Nanotechnology‐derived foods have a significant possibility of broadening the number of options for the formulation and manufacture of functional meals. In the future, nanotechnology may eventually come to dominate the whole area of food production.

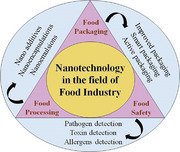

## 1 Introduction

Nanotechnology is an upcoming revolution that promises enormous benefits across all industries, from manufacturing to medical to the food production sector. In this field, at least one fundamental dimension is measured in nanometers, often between 1–100 nm.^[^ ^1^ ^]^ It focuses on nanoscale resources and has the potential in making new and innovative devices and methods. Nanoparticles are thought to be the smallest objects that may operate as a single entity, displaying novel behaviors and characteristics.^[^ ^2^ ^]^ Since nanoparticles have a larger surface area and mass transfer rates than big particles (of the same composition), they seem to have higher biological and chemical activity, penetrability, catalytic behavior, enzymatic reactivity, and quantum characteristics.^[^ ^3^ ^]^ The dimensions, construction, and properties of nanomaterials are used to classify them into several categories. Nanomaterials with a high surface‐area‐to‐volume ratio (SA:V) have the potential to exhibit desirable physiochemical characteristics, including those related to diffusivity, solubility, color, thermodynamics, optics, and magnetization.^[^ ^4^ ^]^ Nowadays, the food market is growing day by day. In 2023, the food market will generate $9.43 trillion in revenue. The market is expected to grow by 6.21% yearly. With a market volume of US$1.64 trillion in 2023, Confectionery & Snacks will be the market's largest sector. According to population estimates, US$1228.00 in income is expected per person in 2023. By 2023, internet sales in the food industry will account for 8.5% of total revenue. In 2023, it is estimated that the Food market would have a volume per person of 340.87 kg. Today, the market of food needs innovations that can make food that is easy to use, real, and delicious so that it can keep its lead in the food processing and distribution sector. One of these is nanotechnology, which has numerous implementations in food sector/market. Due to their ability to prevent spoilage, nanoparticles are increasingly being employed in the food industry. Preservatives, antimicrobial sensors, flavoring agent, wrapping materials, packed food components, and so on are all examples of nanomaterials and nanoscale food additives used to modify nutritional profiles and enhance product longevity, quality, appeal, etc.^[^ ^5^ ^]^ Nanotechnology enables the production of novel products and the growth of applications in food systems, including functional foods, pharma foods, nutraceuticals, bio‐actives, and others. This potential may be multiplied via the use of nanotechnology.^[^ ^6^ ^]^ It has even been shown to be useful for identifying viruses in food, which may then be used as biomarkers of food safety and quality.^[^ ^7^ ^]^ When food is processed, a method called nanoencapsulation may be used to encase nano‐sized food components, additives (such as taste and color), and nutritional supplements (such as proteins and antioxidants), which can then be included into functional foods. This improves transport and disposability for insoluble water substances (can be made soluble by formulation of nanoparticles), masks unusual tastes and odors, and creates protective barriers and controlled release.^[^ ^8^, ^9^ ^]^ It is also a goal of nanotechnology to combat food‐related disorders, such as obesity and diabetes; design appropriate nutrition diets for various specific categories, lifestyles, and aging populaces; and ensure that food production is as environmentally friendly as possible.^[^ ^10^ ^]^ This innovation permits the development of nutrition nano‐therapy gadgets with extreme accuracy. It has the potential to create smart/intelligent systems for the nano‐encapsulated, regulated nutrient release. Nanoscale enzymatic reactors may be created, allowing for the growth of fresh foodstuffs through the use of the fortification of food.^[^ ^11^ ^]^ Widespread interest is being shown in electro‐spun nanofibers as potential food‐packing materials (like structured polymeric film) or materials for encapsulation.^[^ ^12^ ^]^ Plastics, glass, metals, and wood and its derivatives are the traditional packaging materials used in the food industry. These are delicate substances. They can be broken by strikes. It has a higher density than the other materials. Its manufacturing and production costs are quite expensive. Nanomaterials used for food packaging provide many benefits such as improved mechanical barriers, detection of microbial contamination, and potentially enhanced bioavailability of nutrients. This is probably the most typical use of nanotechnology in the food and allied sectors. This publication represents in‐depth discussion of nanomaterial manufacturing methods and their uses in food. Future possibilities of nanotechnology and the accompanying safety concerns and economic considerations have also been discussed in detail.

## 2 Synthesis of Nanomaterials

**Figure 1:**
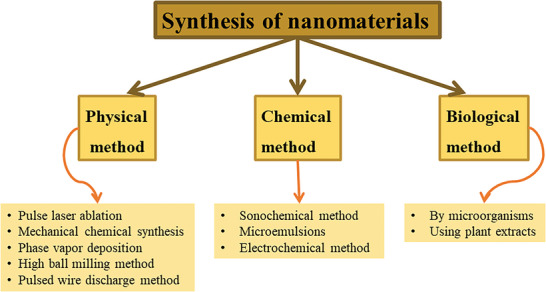
Various processes for nanomaterials synthesis.

Particles, thin films, colloids, tubes, clusters, rods, powders, wires, and many other forms of nanomaterials may be synthesized using a wide range of approaches.^[^ ^13^ ^]^ There are essentially three distinct ways to go about producing a nanomaterial, and they are shown in **Figure** **1**. The created technique takes into account the material of interest as well as the specific nano‐structures being studied, such as nanoplates, nanorods, nanowires, and quantum dots (QDs).^[^ ^14^, ^15^, ^16^, ^17^ ^]^

### 2.1 Physical Method

The physical approach typically involves the use of evaporation and mechanical forces to synthesize nanomaterial. Examples of physical techniques for synthesizing nanomaterials include mechanical chemical synthesis, pulse laser ablation, wire discharge technique, physical vapor deposition with consolidation, mechanical ball milling approach, and many more.^[^ ^18^ ^]^

#### 2.1.1 Pulse Laser Ablation Method

**Figure 2:**
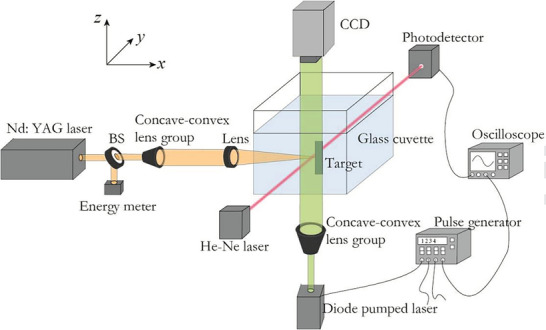
Institutionalized Procedures for Experimentation. The ablation laser beam (yellow) is a Nd: YAG laser moving in the x direction, the optical probe beam (red) is a He‐Ne laser moving in the y direction, and the illuminating beam (green) is a diode‐pumped laser moving in the z direction. (Reproduced with permission.^[^ ^21^ ^]^ Copyright 2017, Elsevier).

In the mentioned technique, plasma is generated by directing a high‐powered pulsed laser beam towards the desired substrate within chamber of vacuum. After the generation of plasma, a colloidal solution of nanoparticles is created.^[^ ^18^ ^]^ As an alternative to a vacuum chamber, nanocomposite‐forming chemicals may be stored in concentrated solutions. Researchers^[^ ^19^ ^]^ used a laser ablation approach to create a copper‐chitosan (Cu‐CS) nanocomposite, and they discovered that the proportion of CS in solution significantly influences the efficiency of nanoparticle production. After incubation for 4 h, they found that the nanocomposite had an antimicrobial effect, significantly slowing the development of a bacterial sample containing 103 bacteria. In addition, the production of antibacterial silver nanoparticles (AgNP) that are used in food packaging material also makes use of the laser ablation technology.^[^ ^20^ ^]^ **Figure** **2** depicts the experimental setup, which combines the optical beam deflection technique with time‐resolved shadowgraphy. X, Y, and Z axes each receive one of three possible beams. The yellow ablation laser beam is directed along the x‐axis, the red optical probe beam along the y‐axis, and the green illumination beam along the z‐axis.^[^ ^21^ ^]^

#### 2.1.2 High Ball‐Milling Approach

**Figure 3:**
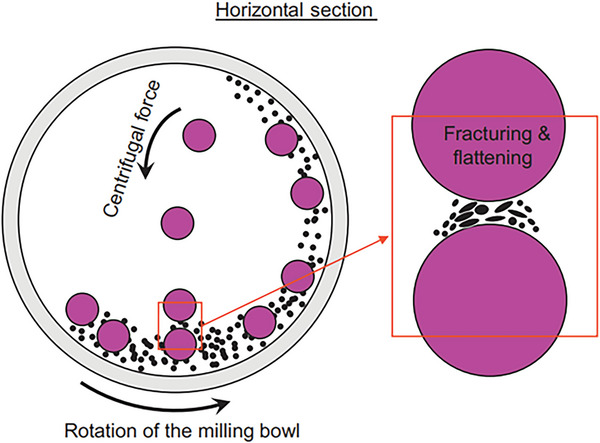
Schematic view of the motion of the ball and powder mixture. (Reproduced with permission.^[^ ^23^ ^]^ Copyright 2018, Elsevier).

The creation of nanoparticles may be accomplished by the use of this method, which involves solid‐state processing technique. At high speeds (a few hundreds of revolutions per minute), containers that hold balls which are made up of tungsten carbide or hardened steel as well as the raw material like flakes or powder (size in microns) are spun around its own axis or some central axis. At this stage, raw material is hurled and then hard‐pressed counter to wall, where it is ground into fine dust particles (between a few nm and few tens of nm).^[^ ^15^ ^]^ There are different kinds of mechanical mills like planetary, tumbler, vibratory, and rod as well as many others that are used commonly. By grinding the material in a ball mill, a variety of substances may be transformed into nanocrystalline forms, including cobalt, chromium, tungsten, Nickel‐Titanium, Aluminum‐Iron, and Silver‐Iron.^[^ ^18^ ^]^ A few of mentioned nano‐particles offer actions that may be employed for active food packaging, which are antiviral, antiyeast, antifungal, and antibacterial.^[^ ^22^ ^]^ The schematic of high ball milling method is shown in **Figure** **3**.^[^ ^23^ ^]^ The role of centrifugal force is very important in the synthesis of nanostructure by this technique.

#### 2.1.3 Mechano‐Chemical Approach

**Figure 4:**
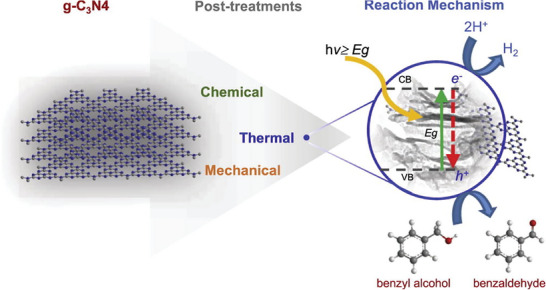
Depicts the mechanism of mechanical chemical synthesis method. (Reproduced with permission.^[^ ^24^ ^]^ Copyright 2017, Elsevier).

Here, mechanical energy, that is, kinetic energy or potential energy or both is applied to trigger a chemical reaction. The precursors are often a combination of metals, chlorides, and oxides. These components undergo a reaction when subjected to grinding or further heat treatment to generate a powder of the composite. This powder consists of ultra‐fine units that are scattered in salt matrix and are stable. These very small particles may be reclaimed by washing them in an appropriate solvent and then selectively removing the matrix.^[^ ^18^ ^]^ The detailed reaction mechanism employed in the mechanical chemical synthesis route involving band diagrams is depicted in **Figure** **4**.^[^ ^24^ ^]^

#### 2.1.4 Pulsed Wire Discharge Technique

**Figure 5:**
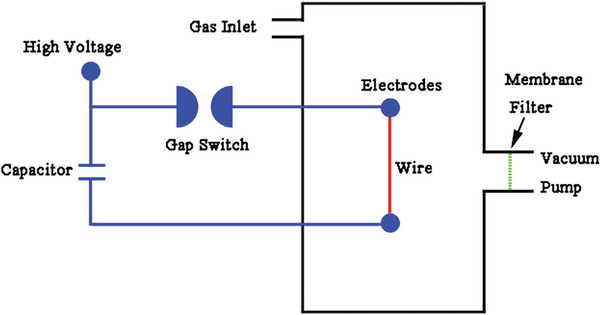
Schematic illustration of the pulsed wire discharge apparatus. (Reproduced with permission.^[^ ^30^ ^]^ Copyright 2014, Elsevier).

This is another physical approach for synthesizing nanoparticles, although its synthesis process is fundamentally distinct from that of other approaches.^[^ ^25^ ^]^ PWD nanoparticle production typically requires a discharging circuit, vacuum system, and powder collection filter. The process involves vaporizing a metal wire by passing a pulsed current across it. The surrounding gas then cools the vapors, causing the nanoparticles’ formation.^[^ ^26^, ^27^ ^]^ Nanoparticles of copper will be created when copper metal is manipulated into a wire shape. It has been stated that PWD can be used to create nanoparticles of oxide, nitride, and metal.^[^ ^1^ ^]^ This technique of producing copper nanoparticles (CuNPs) may be utilized to extend the freshness and firmness of tomatoes, hence extending their storage time. Tomatoes of higher quality for human consumption are reportedly generated after CuNP treatment increases bioactive substance accumulation like lycopene, total phenols, flavonoids, and vitamin C.^[^ ^28^ ^]^ Despite its excellent energy efficiency and potential output rate, the PWD technique is unsuitable for industrial application because of its prohibitively high cost and limited applicability to a wide metallic range.^[^ ^29^ ^]^ Pulsed wire discharge apparatus utilizing Membrane filter, vacuum pump, Gap switch, etc. are shown in **Figure** **5**.^[^ ^30^ ^]^

#### 2.1.5 Physical Vapor Deposition (PVD) with Consolidation

**Figure 6:**
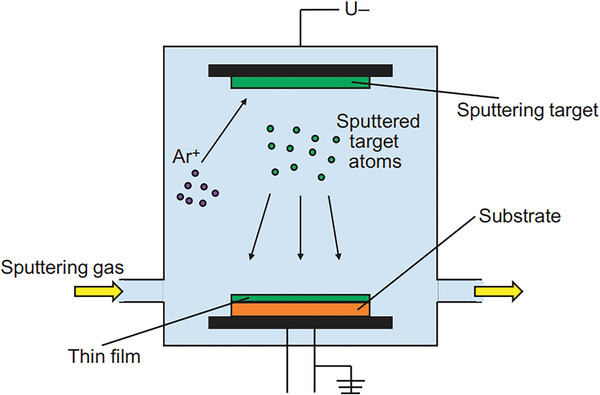
Schematic illustration of the physical vapor deposition process. (Reproduced with permission.^[^ ^23^ ^]^ Copyright 2018, Elsevier).

Here, raw material is vaporized first and after it is slammed into a cloud of reactive gas or inert gas. The scraper is used to remove the nanoparticles that have accumulated on the cold finger. The powder of nanoparticle is then squished by the piston‐anvil. The use of a vacuum chamber for all of the procedures ensures that the final product is as pure as possible.^[^ ^15^ ^]^ Aluminized film suitable for food packaging has been developed using the pulsed vapor deposition method.^[^ ^31^ ^]^ Physical vapor deposition technique utilized in the synthesis of nanomaterials is depicted in **Figure** **6**.^[^ ^23^ ^]^

### 2.2 Chemical Method

Low‐temperature production, easy approach, the probability of the nanoparticles of different shapes and sizes, effortless conversion to thin films or dry powder of the liquid end product, foreign atoms’ absorption during production, and so on are just a few of the benefits of chemical methods over physical methods.^[^ ^16^, ^32^ ^]^ Some examples of chemical techniques are discussed here. These include microemulsion/colloidal procedures, sono‐chemical techniques, electrochemical techniques, and others.^[^ ^18^ ^]^

#### 2.2.1 Microemulsion/Colloidal Technique

**Figure 7:**
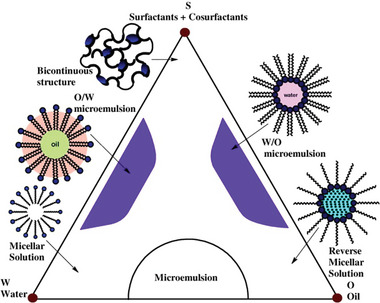
Hypothetical phase regions of microemulsion systems. (Reproduced according to the terms of the Creative Commons Attribution CC BY License.^[^ ^34^ ^]^ Copyright 2012, The Authors, published by Elsevier).

In micro‐emulsion technique of nanoparticle formation, a suitable surfactant is utilized to create a thermodynamically steady dispersion of two immiscible solutions, like water in supercritical carbon dioxide (W/SC‐CO_2_) or oil in water or water in oil.^[^ ^33^ ^]^ Hydrophobic surfactants in nanoscale micelles and oils aim toward the aggregate's core, whereas hydrophilic head groups face the water that serves as the bulk solvent.^[^ ^18^ ^]^ Microemulsion systems with hypothetical phase regions are shown in **Figure** **7**.^[^ ^34^ ^]^

#### 2.2.2 Sono‐Chemical Method

**Figure 8:**
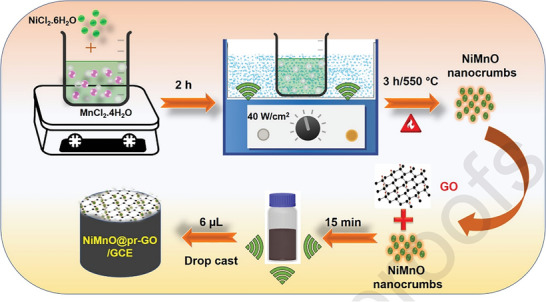
Sonochemical synthesis of NiMnO@pr‐GO nanocomposite. (Reproduced with permission.^[^ ^38^ ^]^ Copyright 2020, Elsevier).

Sono‐chemical process involves the application of high‐intensity ultrasonic radiations (in the range of 20 kHz–10 MHz) to molecules that generate acoustic cavitation with the purpose of nanoparticle synthesis. Ultrasonic pulses and electrolytes both are used in the process of solo electrochemical production in order to produce nanoparticles.^[^ ^18^ ^]^ The method is easy to implement, can be performed under ambient conditions, and allows for simple size regulation of nanoparticles by varying precursor quantities.^[^ ^35^ ^]^ In order to create silver nanoparticles, a sono‐chemical process is performed using gelatin as a stabilizer.^[^ ^36^ ^]^ Silver nanoparticles like this may be used as antimicrobials in food packaging as well as other coatings.^[^ ^37^ ^]^ The detailed overview of the synthesis of nanomaterials using sono‐chemical synthesis is shown in **Figure** **8**.^[^ ^38^ ^]^

#### 2.2.3 Electrochemical Method

**Figure 9:**
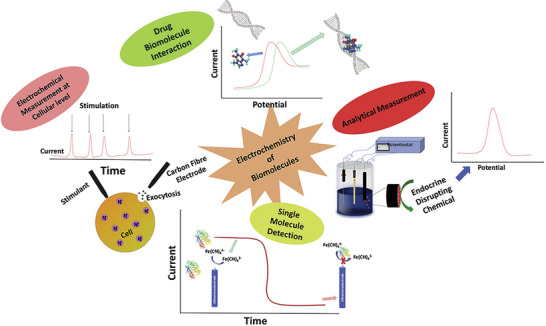
Detailed analysis of electrochemical method. (Reproduced with permission.^[^ ^43^ ^]^ Copyright 2022, Elsevier).

In this scenario, the electrolyte is between two electrodes, and an electric current is carried between them. At the contact between electrode and electrolyte, electricity acts as the driving or controlling force, causing the formation of the nanoparticles. The benefits of this technology include not requiring a vacuum system, having minimal expenses, having a simple operation, having a high degree of flexibility, having reduced contamination (resulting in pure products), and having an environmentally safe process.^[^ ^18^ ^]^ Using a concentration of about 0.01 mm solution of silver nitrate AgNO_3_, a glassy carbon electrode as the working electrode, and silver metal as the counter electrode, the electrochemical technique can generate nanoparticles of silver with a size not larger than 20 nm.^[^ ^39^ ^]^ Electrochemistry was used to create the silver nanoparticles, which, in contrast to Gram‐negative bacteria, had a bactericidal action against Gram‐positive bacteria.^[^ ^40^ ^]^ The synthesis of high‐quality nanoparticles whose dimensions can be modified by changing the current density using the electrochemical approach is the method's key benefit. Also, this process does not need the use of costly equipment or a vacuum.^[^ ^41^ ^]^ Nevertheless, there are a few limitations, like the deposition of silver (Ag) on cathode, which lowers the effective surface area for particle creation throughout the electrochemical production of the silver nanoparticles. This is one of the limitations. In addition to this, there is a possibility that the formation of nanoparticles will be halted if the whole region is coated by electrodeposits of silver.^[^ ^42^ ^]^ Each and every parameter involved in the electrochemical method with differential pulse voltammetry is shown in **Figure** **9**.^[^ ^43^ ^]^

### 2.3 Biological Method

Synthesizing nanomaterial using biological processes provides several benefits, including nontoxicity, ease of augment, repeatability in manufacturing, symmetrical shape, environmentally‐safe procedures, and a lot more. The creation of nanoparticles by microorganisms like yeasts, fungus, bacteria, and other creatures like these, as well as the synthesis of nanoparticles by plant extracts, are two of the biological techniques of eating nanoparticles that are addressed more below.^[^ ^44^ ^]^

#### 2.3.1 Nanoparticle Synthesis from Microorganisms

**Figure 10:**
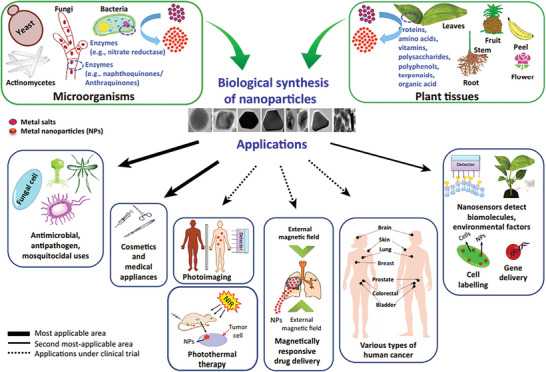
Biological Synthesis and Applications of Metal Nanoparticles in Biomedical and Environmental Fields. (Reproduced with permission.^[^ ^47^ ^]^ Copyright 2016, Elsevier).

The synthesis of NPs by a living organism begins with the microorganism capturing certain metal ions from their surrounding environment and then transforming those metal ions into elemental metal using enzymes that were created as a byproduct of the cell's metabolic processes. On the basis of the site where nanoparticles are generated, synthesis may be classified into two types extracellular and intracellular. In the presence of enzymes, ions can be converted into nanoparticles both on surface of the cell as well as inside the microbial cell during extracellular and intracellular processes, respectively.^[^ ^17^ ^]^ These nanoparticles include sulfide nanoparticles, metallic nanoparticles (silver, alloy, gold, etc.), and oxide nanoparticles (nonmagnetic as well as magnetic). Nanoparticles like this may be utilized as drug carriers for direct target medication delivery, biosensors, antibacterial agents, response rate enhancers, and many more applications.^[^ ^45^ ^]^ There have been reports of nanoparticles like zinc oxide (ZnO) being used as antimicrobial agents and food additives. It can be manufactured utilizing reproducible bacteria such as *Aeromonas hydrophila*.^[^ ^46^ ^]^ The detailed layout for the synthesis of nanoparticles through biological methods having diversified applications is shown in **Figure** **10**.^[^ ^47^ ^]^

#### 2.3.2 Nanoparticles Synthesis from Plant Extracts

**Figure 11:**
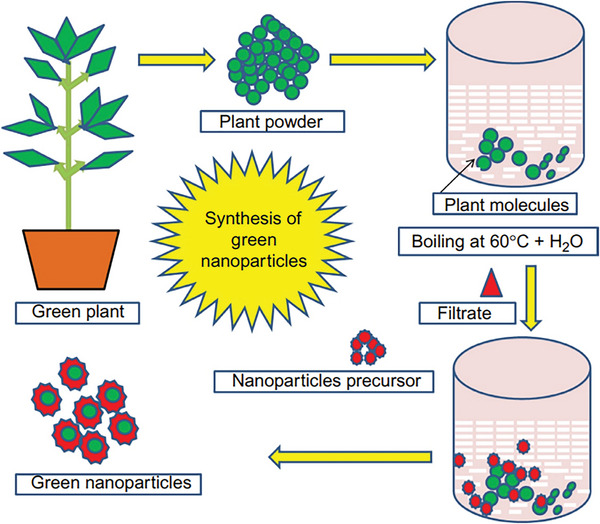
Pictorial representation of the method of green nanoparticle synthesis. (Reproduced with permission.^[^ ^54^ ^]^ Copyright 2019, Elsevier).

The several components of the plant, including the fruits, leaves, roots, stems, and extracts of those parts, are all viable options for the production of metal nanoparticles.^[^ ^48^ ^]^ The process of production might involve the lessening of metal salt together with reducing and stabilizing agents by proteins, amino acids, enzymes, organic acid, and vitamins, in addition to secondary metabolites, like alkaloids, flavonoids, heterocyclic compounds, polyphenols, terpenoids, and polysaccharides that are in the plant parts.^[^ ^49^ ^]^ Some examples of these nanoparticles include copper, silver and titanium dioxide, cadmium, silver and gold. Some of the many uses for these nanoparticles include their catalytic, antibacterial, electrocatalytic, cytotoxic, luminescent, and electrocatalytic actions toward hydrogen peroxide, etc.^[^ ^44^ ^]^ Antibacterial efficacy and potential use in food packaging were reported for silver nanoparticles made by the reduction and stabilization of silver ions with a mixture of biomolecules derived from plant extract.^[^ ^50^ ^]^ In addition to their own advantages and disadvantages, the various nanoparticle synthesis techniques also have their own distinct strengths. However, nanoparticles are often employed to increase the antimicrobial impact of food packaging material, making them a popular choice for this purpose. In this context, AgNPs are the nanoparticles of choice because of their antimicrobial effectiveness. Therefore, electrochemical approach, sono‐chemical technique, high ball milling technique, and pulse laser ablation method may be regarded as the commonly studied and utilized procedures to make antibacterial wrapping materials for food industry.^[^ ^40^, ^51^, ^52^, ^53^ ^]^ Other than antibacterial packaging, nanoparticles like CuNPs made using the PWD approach have been used to increase the shelf‐life of food by making it denser, namely tomatoes.^[^ ^28^ ^]^ The overview of green synthesis of nanoparticle is demonstrated in **Figure** **11**.^[^ ^54^ ^]^ **Table** **1** provides the advantage and disadvantage of each method. From Table 1, it is concluded that the chemical methods for the synthesis of nanoparticles are preferred because of its advantages over other methods. Specially in chemical methods, electrochemical method is preferred because a variety of nanomaterials are synthesized by this process.

**Table 1:** Advantages and disadvantages of various synthesis methods

Method	Types	Advantages	Disadvantages
Physical Method	Pulse laser ablation	eco‐friendly way, can be conducted in vacuum or in ambient media (gas or liquid)	exposure to radiation, require high energy and temperature
	Mechanical chemical synthesis	large‐scale production, controlled morphology, fast process	thin film formation is not possible, limited to only nanoparticles
	Phase vapor deposition	no use of toxic chemicals, purity	cooling systems are required, require high temperature
	High ball milling method	Cost‐effective, can be used for both wet and dry grinding processes	noisy machine, slow process, difficult to clean
	Pulsed wire discharge method	High speed, use of exotic materials, uniform size and shape.	Productivity, high cost, alter surface chemistry and physicochemical properties
Chemical Method	Sonochemical method	Non‐hazardous, rapid in reaction rate, high versatility in surface chemistry, easy functionalization, size controllability	Amorphous nanoparticles are formed
	Microemulsions	easily prepared, thermodynamically stable.	limited solubilizing capacity for substances with high melting points
	Electrochemical method	Cost‐effective, robust, and in most cases, it's a green approach	use of toxic chemicals and organic solvents
Biological Method	By microorganisms	ecofriendly, nontoxic and biocompatible	Hard to control size, shape, and crystal growth. Possible presence of endotoxin
	Using plant extracts	Simple, facile ecofriendly, cost‐effective, and nontoxic	time‐consuming purification processes.

## 3 Nanotechnology in Food Packing and Security

**Figure 12:**
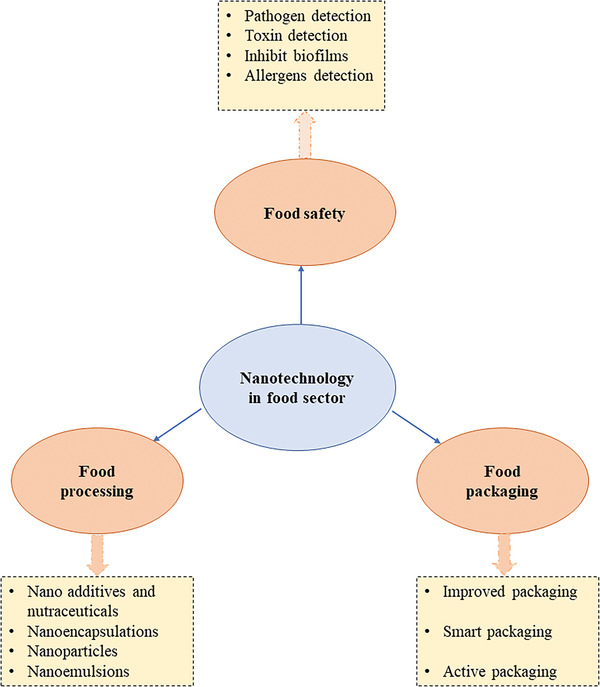
The applications of nanotechnology in safety, packaging, and processing of food.

Among many processes necessary to ensure the safety of food, the packaging is vital. No packaging material is 100% impermeable to elements including natural chemicals, atmospheric gases, and water vapors.^[^ ^55^, ^56^, ^57^ ^]^ However, it is not ideal to completely prevent the movement and permeability of gases when it comes to the packing of fresh vegetables and fruits since these foods go through a process called cellular respiration.^[^ ^56^ ^]^ In comparison, carbonated drinks shouldn't be left exposed to air or carbon dioxide (CO_2_) in their container. This is done to avert decarbonation and oxidation.^[^ ^56^ ^]^ Dependent on the food matrices and the types of packaging materials used, the exchange of oxygen (O_2_), water vapor (H_2_O), and carbon dioxide (CO_2_) take place in different ways. Because of this, different nanocomposite materials, such as polymers, may be utilized to report and solve these difficulties in packaging of food.^[^ ^58^ ^]^ Nanoparticles with a diameter of less than 100 nm are about one hundred times thinner than the thickness of an approx. 10 000 nm human hair, and it is a thousand times thinner than the thickness of an approx. 100 000 nm book page. It is possible for various nanoparticle structures to be of significant use in a variety of areas of the medicinal and food industries, particularly in research programs that are connected to food science, provided that an appropriate regulatory processing approval is obtained (**Figure** **12**). Recent advances in the utilization of nano‐bio composites in food packing have resulted in an improvement in the capacity of food packing to serve as a hurdle contrary to the accumulation of gases.^[^ ^59^ ^]^ **Table** **2** provides a concise overview of the several nanoparticle uses that may be found in food packaging. Recent developments in the industry of food packing are promoting the usage of decomposable polymers that are supplemented with environmental friendly nano‐fillers.^[^ ^60^ ^]^ On the other hand, there is a significant cause for worry over the intake of these nano‐compounds while the meal is being consumed. As a result, it is very crucial to conduct studies on the migration of these nanoparticles throughout the human body in addition to the immunogenic and toxic effects of these nanoparticles.^[^ ^61^ ^]^ Concerns have also been raised about the biological decomposability of these nano‐filled decomposable polymers.^[^ ^62^ ^]^ Scientists looking for nanomaterials that are safe for humans and the environment take these concerns very seriously.^[^ ^63^, ^64^ ^]^

**Table 2:** Nanoparticles for application in food packaging

Types of nanoparticles	Methods of synthesis	Matrix	Application	Reference
Zinc oxide	Chemical & Biological	Orange juice, Liquid egg albumen	Effectively reduces *Lactovacillus plantarum*, *Samonella*, yeast, and mold counts without changes in quality parameters.	[65, 66, 67]
Titanium oxide	Chemical	Chinese jujube, Strawberry	Reduces browning, slow‐down ripening, senescence, and decay	[68]
Silver oxide	Biological	Apple slice	Retards microbial spoilage	[69]
Silver	Biological	Asparagus, Orange juice, Poultry meat, Fresh‐cut melon, Beef meat exudates	Retards the growth of *aerobic psychrotrophics*, yeasts, and molds; antimicrobial effect against *Escherichia coli* and *Staphylococcus aureus*	[65, 70, 71, 72, 73]

### 3.1 Nano‐Coatings as Intelligent Packing for the Surfaces

Nano‐structured materials, when combined with biopolymers, may either enhance the feature attributes of the unmodified polymer or boost the functional qualities of intelligent and active food packaging. Better‐quality packaging, lively packaging, and intellectual packaging all describe different kinds of packaging that have different potential uses.^[^ ^74^ ^]^ But the European Union (EU) has officially banned the usage of “active” also “intelligent” packing materials in food, with the exceptional use of titanium nitride (TiN) in plastic bottles.^[^ ^75^ ^]^ Mill^[^ ^76^ ^]^ made a nanoparticle tin dioxide or titanium dioxide (TiO_2_) particle‐based desirable photo‐indicator intelligent ink that used a redox‐activate methylene blue dye for oxygen detection in packaging. This detector could slowly change color in reaction to small variations in the amount of oxygen. In previous years, packing industry has paid more attention to nanostructures and certain nanomaterials, such as nano‐clay particulates, because they are easy to get, cheap, easy to work with, and work well. Positive progress has also been made with other nanomaterials that are carbon‐based, such as carbon nano‐tubes and graphene nano‐sheets.^[^ ^77^ ^]^ Since polymeric substrate‐based static or flexible films and bottles (made from a variety of materials together with plastic and glass etc.) with vacuum‐coated thin inorganic layers are effective at blocking oxygen and odors, they are in high demand in the material packaging industry. Although aluminum foil and aluminum‐metallization were traditionally employed, newer materials are increasingly being used. The term “nanocoating”^[^ ^78^ ^]^ is used to describe uniformly thick layers (10 to 100 nm). A small group of scientists has, in recent years, created a nanocoating film with the intelligence to reveal whether or not contamination happened while being stored. Gas concentration and non‐invasive recognition techniques have also shown prodigious capability to continuously as well as effortlessly observe the excess moisture, gas content, and oxygen concentration of a package's headspace, offering effective methods to analyze the food's safety and quality even after the construction procedure.^[^ ^79^, ^80^ ^]^ Oxygen's potential to establish a suitable atmosphere for microbial development within the container might affect the food's lifespan.^[^ ^81^ ^]^ In situ silica nanocomposite film created by Yu et al.^[^ ^82^ ^]^ significantly reduced moisture permeability by 10.2% and oxygen permeability by 25.6%, indicating that films prolonged the safeguarding life of cherries by threefold compared to usual packing. To contest the growth of bacterial biofilms on food packaging effectively, Swaroop and Shukla^[^ ^83^ ^]^ combined nanostructured magnesium oxide (MgO) with polylactic acid (PLA) biopolymer. For the same reason, Foltynowicz et al.^[^ ^84^ ^]^ produced zerovalent Fe particles to serve as oxygen scavengers in food packing.

### 3.2 Nanoparticles as Antimicrobial Agents in Active Packaging

To a certain degree, our food items have been safeguarded against foodborne disease outbreaks caused by the ingestion of damaged packaged food owing to the amazing usage of nanoparticles with antibacterial capabilities. Active food packaging, in contrast to traditional food packaging, not only acts as a passive barrier, but also removes certain unfavorable elements, such as air or water vapor, and aids in the release of antioxidant and antibacterial substances by direct contact with the food. In most cases, these interactions enhance food durability.^[^ ^85^ ^]^ Certain molecules inside the active packaging may either absorb or release the ingredients within the food or the environment outside the box, respectively. Currently, antimicrobial packaging has been the primary focus for the development of active polymer nanoparticles for use in food packaging.^[^ ^86^ ^]^ Overall, numerous bioactive chemicals may be included into the packing substance by capping the substance with nanoparticles, capsulation, or applying other nano methods, improving the performance and efficacy of packaged food.^[^ ^87^ ^]^ Choosing an effective antimicrobial material based on a unique technique to assure the quality of the packed items, including their sensory levels and, visual appearance is essential for the successful implementation of antimicrobial active packaging for food products.^[^ ^88^ ^]^

It is vital for the bioavailability and safeguarding of bioactive components to encapsulate nutraceuticals and functional antimicrobial compounds. This is because encapsulation is particularly significant in food storage, food processing, and transit through the gastrointestinal system. The nanoparticles made of macromolecules that were found in food not only made bioactive polyphenolics like curcumin, *epigallocatechin‐3‐gallate*, and resveratrol more bioavailable, but then they also improve the solubility of these polyphenols and, as a result, help deter their degradation in the environment of the gastrointestinal tract. Spray cooling,^[^ ^89^ ^]^ coacervation,^[^ ^90^, ^91^ ^]^ nanoemulsion,^[^ ^92^ ^]^ extrusion,^[^ ^93^ ^]^ fluidized bed coating,^[^ ^94^ ^]^ and spray drying^[^ ^95^ ^]^ are just a few of the encapsulation methods that have been utilized to create nano or micro‐particulate systems. Foods that have been nano‐encapsulated may have nanoparticles in them without the consumer having to worry about coming into touch with them. Silicon dioxide (SiO2), as one well‐known example, is extensively used as an aroma carrier in many different types of food items.^[^ ^96^ ^]^ Numerous lipid‐based nano‐encapsulation methods have been developed to improve the antioxidant action of the individual constituents by increasing their bio‐availability and solubility^[^ ^97^ ^]^ as well as their targeted, site‐specific delivery with efficient absorption.^[^ ^98^ ^]^ Nanoparticle edible coatings have been shown to be an efficient method of preserving food, extending its shelf life, and protecting it against decomposition by microorganisms.^[^ ^99^ ^]^ The fresh quality foods may be preserved throughout long‐term storage by using coatings created with gelatin that includes nanocrystals of chitosan with nano‐silica,^[^ ^100^ ^]^ cellulose,^[^ ^101^ ^]^ lysozyme or nano‐laminate alginate,^[^ ^102^ ^]^ chitosan, or nano‐silica.^[^ ^103^ ^]^ Nano‐packaging, which uses merged polyethylene with nano‐powders like kaolin, silver, rutile TiO_2_, and anatase TiO_2_, is a unique and simple way for preserving fruits like strawberries (*Fragaria ananassa Duch*.).^[^ ^104^ ^]^ In spite of many studies in nano‐encapsulation using a wide range of materials,^[^ ^105^ ^]^ the appropriate distribution of products and their safety has not been well‐researched. Thus, the enduring poisonousness of nano‐encapsulated meals has to be explored in the future.^[^ ^106^ ^]^ In complement to this, Johnston^[^ ^107^ ^]^ utilized nano‐structured calcium silicate for the absorption of Ag^+^ ions from a solution. This resulted in the formation of NCS‐Ag complex, which was then effectively utilized as an antimicrobial agent for the purposes of food packing. In a similar vein, titanium dioxide (TiO_2_) is often employed in coatings of surface as a photocatalytic disinfection material^[^ ^108^ ^]^ and to neutralize certain harmful bacteria present in food.^[^ ^109^ ^]^ The interface between the negatively charged cell membranes and the positively charged chitosan may be the mechanism by which chitosan‐encapsulated nanoparticles exert their antibacterial activity. Through these interactions, membrane permeability is increased, leading to a breakdown and the subsequent release of intracellular contents.^[^ ^110^ ^]^ Films made from nanocellulose, polyvinyl alcohol (PVA), and silver nanocomposite were used for antimicrobial packaging of food by Sarwar et al.^[^ ^111^ ^]^ Both the Methicillin‐resistant Staphylococcus aureus (MRSA) and the DH5‐alpha strain of Escherichia coli are effectively countered by these films’ potent antimicrobial properties. Nanostructured, Al‐doped ZnO‐based, PLA‐functionalized antimicrobial layers were created by Valerini et al.^[^ ^112^ ^]^ employing sputtering power. Significant antibacterial potential against *E. coli* was observed, and the coatings were suggested as viable materials for ecological active packing. Also, Lu et al.^[^ ^113^ ^]^ created a 100 nm antimicrobial nano‐emulsion by encapsulation of citral essential oil with ultrasonic power; the proposed nano‐emulsion was very effective in providing an antimicrobial agent (citral essential oil) in the food industry. The antimicrobial properties of nano‐composites may be enhanced by active packaging options based on metal oxide metal and nanoparticles (NPs). Silver (Ag) nanoparticles are among the most commonly employed types of metal nanoparticles in industries. Because silver nanoparticles significantly boost the permeability of the cell membrane through surface of cell adhesion and by degradation of lipopolysaccharide,^[^ ^114^ ^]^ they are harmful for a variety of food pathogens.^[^ ^115^ ^]^ Once inside the bacterial cell, they can harm the DNA^[^ ^116^ ^]^ as well as release antimicrobial silver ions,^[^ ^117^ ^]^ that bind to nitrogen, oxygen, or sulfur comprising electron donor groups in molecules thereby preventing DNA replication and ATP synthesis. When exposed to mm concentrations of Ag^+^ ions, the cytoplasm shrinks, the cell wall membrane separates, peptidoglycan in cell wall is destroyed, the ribosomes are denaturated, and DNA is compressed, all of which inhibit DNA formation, rupture cell membrane, and eventually cause the death of cell.^[^ ^118^ ^]^ The three key mechanisms of the harmfulness of metal‐containing nanoparticles to bacteria that are generally known to exist: the production of reactive oxygen species (ROS), which causes oxidative cellular impairment, the disruption of the bacterial membrane, and the uptake of metal ions, which depletes intracellular ATP. Reports have also verified the immobilization, inclusion, layering, and surface alteration of antimicrobial components on top of packing material.^[^ ^119^ ^]^ In addition, direct insertion of antimicrobial components into packaging sheets allows for the achievement of a greater antimicrobial effect. Similarly, films that have been coated with a variety of efficient antimicrobial components might have a greater antimicrobial potential. Volatile and non‐volatile antimicrobial chemicals may be included into film packaging to be spread onto food surfaces by evaporation, migration, or diffusion.^[^ ^120^ ^]^ A bio nanocomposite film made from silver copper bimetallic nanoparticles and fish skin gelatin was recently developed by Arfat et al.^[^ ^121^ ^]^ Both *Listeria monocytogenes* and *Salmonella enterica* were eliminated by the film, demonstrating its strong antibacterial potential. The growth of *Campylobacter, E. coli*, and *Salmonella* has been slowed with the use of a number of antimicrobial master batches based on silver, including *Bactiblock*, *Surfacine*, *Aglon*, *Irgaguard*, *IonPure*, and *Biomaster*.

## 4 Food Packaging Methods

The aim of food packing is to sustain the food's freshness while also guaranteeing that it will not spoil before being consumed. Physical protection is the primary goal of food packaging, which also includes preventing deterioration by scavenging spoilage‐causing gases like oxygen and shielding food from external shocks and vibrations as well as microorganisms and extreme temperatures. Using biodegradable materials for packaging helps lower pollution levels. With the advent of nanotechnology in the food packaging business, this concept has become a practical reality. Careful consideration must be given to the use of high‐barrier plastics, the incorporation of antimicrobials, and the implementation of contamination identification techniques when packing food.^[^ ^122^ ^]^ Thus, food preservation refers to the processes used to keep food from spoiling by eliminating the growth of microorganisms that may otherwise compromise its flavor, texture, and nutritional value. Some conventional food preservation techniques include drying, canning, and freezing. There are several processes involved in managing food, including processing, packing, and preservation methods. Conventional materials used in food packaging are metal, paper, glass, and plastic. There are many different types of metal‐based food packaging products in the market, including cans, containers, and caps. Modern manufacturers employ recyclable materials that are covered with organic material to prevent food from coming into touch with metal. The choice of metal depends on its rigidity and strength, blocking moisture, temperature‐tolerance, and corrosion protection. The drawbacks of metal as a packaging material are its high cost, inability to sustain corrosive compounds, and ductility which might impede shipment. Paper is one of the earliest packing materials still in use today. Corrugated boxes, milk/folding cartons, cups, bags, tubes, sacks, labels, paper plates, pamphlets, and wrapping paper are examples of materials that are often used. Features of paper packing that are advantageous: Paper easily shreds along the fibers, and folding is simplest from end to end. Across all fibers, fold durability is greatest, and rigidity level is good (cardboard). Paper has a few drawbacks as a packaging material, including its limited resistance and the requirement for additional materials when containing food. Another long‐used, widely used packing material is glass. In 7000 B.C., the earliest traces of glass production were discovered. Glass is now one of the most trustworthy and non‐toxic materials for food and beverage packaging. Glass is made by melting soda, lime, and silica at extremely high temperatures, followed by construction of containers. Other additional ingredients may be added depending on the required properties. Glass is favored as a packing material because of its inertness, sterilizability, tamper‐proof, and microwaveable characteristics. Glass has a number of drawbacks that make it undesirable for use in packaging, including its fragility, the possibility of breakage in the event of a blow, its weight relative to other materials, and the high cost of manufacture and processing. Another common material for food packing is plastic which is broadly used in bowls, pots, bottles, trays, foils, cups, bags, and pouches. The positive aspects that work in its favor are its lightweight design and relatively reduced cost. Plastic is preferred as a good option for food packaging as it is lightweight, and can be molded into infinite number of forms. Its drawback of using plastic as a packaging material is that extreme heat might cause it to melt or distort. It is not biodegradable and has a significant negative environmental impact.

Nanomaterials are favored to solve these issues that emerge with traditional materials. When utilized for food packaging, nanomaterials provide a number of advantages, including greater mechanical barriers, microbial contamination detection, and maybe increased nutritional absorption. The food business uses a variety of nanocomposites, polymers incorporating nanoparticles, for food contact materials and packaging. Nanosilver, nano‐titanium dioxide, nano‐magnesium oxide, nano‐copper oxide, carbon nanotubes, etc. are a few of them. Utilizing package components that interact with food, the environment, and both, active packaging actively extends the product's shelf life.

**Figure 13:**
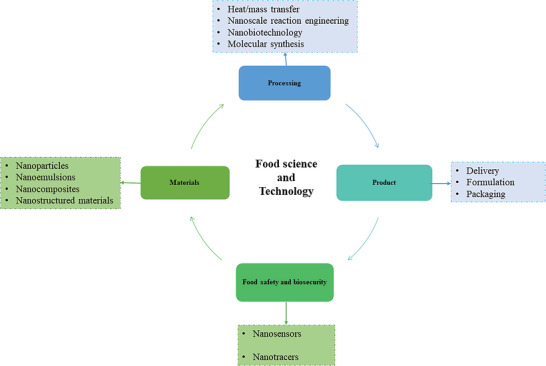
Various stages of food management and the use of nanotechnology.

Nanotechnology, with the aid of a variety of nanomaterials, facilitates each of these procedures (**Figure** **13**).^[^ ^122^, ^123^ ^]^

### 4.1 Edible Thin Film Packaging

Eatable thin film or packaging may extend the freshness and quality of perishable foods by preventing oxidation. Materials used to create bioplastic for use in edible thin film packaging include chitosan, carrageenan, poly‐lactic acid, gelatin, blends of starch and sodium caseinate, alginate, poly‐glycolic acid, and many more. The films behave as active packing, growing barrier protection, avoiding gases like ethene (C_2_H_4_) and oxygen from destroying food substances and conserving product's impression.^[^ ^124^ ^]^ They have a wide range of potential applications, including protecting fruit, meats, vegetables, candies, chocolates, French‐fries and baked goods. Edible nano laminates might be used to create a thin layer that would serve to preserve the food by blocking out any unwanted elements such as moisture, lipids, gases, and off‐flavors or aromas. Polysaccharides, proteins, and even lipids may all be used to create these substances. While the polysaccharide and protein films are effective at blocking both carbon dioxide and oxygen they are not as effective at blocking moisture. Lipid films are effective for this purpose, despite their low mechanical strength and lack of resistance to gases. Nano laminates may be made by combining functional compounds like antimicrobials, antibrowning factors, antioxidants, colors, flavors, and enzymes with edible films. Chitosan and sodium alginate, two polysaccharides with opposing charges, were placed onto amino lysed/charged substrates to create a nano‐layered film.^[^ ^124^ ^]^

### 4.2 Nano Encapsulation

Nanocapsules are used to perform the process of nanoencapsulation. The advantages they offer include improved bioavailability and efficiency, as well as convenience in handling, increased stability, protection from oxidation, retention of volatile substances, sensory attributes enhancement, controlled release based on moisture or pH, sequential distribution of numerous active ingredients, a shift in flavor character, and prolonged organoleptic perception. They are nano vesicular systems, and they have the classic core‐shell architecture, with the drugs enclosed in a reservoir or cavity and protected by a polymer membrane or coating. The active agent may exist in the cavity as a liquid, a solid, or a molecular dispersion. The storage of food is facilitated by nanocapsules, which play a role in both the supply of the required ingredient and the trapping of odor and undesired substances in the meal.^[^ ^122^ ^]^ Food supplements are transported by nanocapsules via the digestive tract, increasing their bioavailability in the body. The five fundamental methods for manufacturing nanocapsules are: a) Emulsion‐diffusion, b) Nano precipitation, c) Layer by layer, d) Double emulsification, and e) Polymer coating. The main distinction between nanoemulsions and traditional emulsions is that nanoemulsions do not alter the food's visual appeal. These tiny capsules are used to provide plant vaccinations and fertilizers. They may also be employed to improve the nutritional value of meals by transporting lipophilic health supplements including minerals, vitamins, growth hormones, and fatty acids. The primary advantage of encapsulation is that the concealed component can be reliably delivered to the intended location even when environmental circumstances are adverse. One kind of nano‐based carrier utilized for nano encapsulation is the liposome. Nano‐liposomes are helpful in achieving regulated and targeted distribution of the various components included within the system. They have been shown to transport a variety of bioactive compounds including minerals, nutraceuticals, enzymes, antimicrobials, vitamins, and additives.^[^ ^125^ ^]^ Gallic acid is being encapsulated into zein fibers via electrospinning, a novel encapsulation technology.^[^ ^126^ ^]^ The zein fiber prevents the lipids from breaking down on the way to the delivery site. The food packaging business may make extensive use of this innovative approach. As a result of the increased specificity and solubility of the components contained by lipid‐based encapsulation techniques, they are much superior to alternative encapsulation systems.

### 4.3 Nano Emulsion

In the food industry, nanoemulsions are employed to create items like flavored oils, salad dressing, sweeteners, individualized drinks, and more. The various stimuli (ultrasonic waves, pH, heat, and so on) aid in the release of a wide variety of tastes. They do an excellent job of preventing the tastes from being altered by oxidation and enzymes, so they may be enjoyed for much longer. Two primary methods are used to produce nanoemulsions; The high‐energy strategy comprises high‐pressure homogenization, ultrasonic technique, high‐velocity liquid coaxial jets, and high‐speed devices method, and in a similar fashion, the low‐energy technique comprises membrane emulsification, solvent displacement, spontaneous emulsification, the emulsion inversion point, and the phase inversion point. The nanoemulsions are produced by dispersing liquid phase in aqueous phase that is continuous. Nanoemulsions are made using lipophilic components that are completely dispersed throughout the oil phase. Many parameters, including molecular and physicochemical features, determine where the lipophilic constituent is located within the nanoemulsion. Hydrophobicity, surface activity, oil‐water partition coefficient, solubility, and melting point are all physicochemical properties. Using nano emulsion creation, several lipophilic components may be encapsulated.^[^ ^122^ ^]^ For instance, citral, β‐carotene, tributyrin, flaxseed oil, coenzyme Q capsaicin, and several oil‐soluble vitamins. Nanoemulsions have superior thermal stability over traditional emulsions and a great stability to gravitational separation and droplet aggregation. Compared to traditional emulsions, nanoemulsions are favored nowadays because of smaller droplets, bigger surface area, and therefore easy digestion and absorption.^[^ ^122^ ^]^

### 4.4 Nano Sensors

Nanosensors can detect a shift in hue as well as the gases released during deterioration. Sulfur dioxide (SO_2_), Hydrogen (H_2_), nitrogen oxides, hydrogen sulfide (H_2_S), and ammonia (NH_3_) are some of the gases to which the sensors are most attuned.^[^ ^122^ ^]^ They are a sensor and processing unit in one, able to convert changes in heat, light, gas, humidity, and chemicals into electrical impulses. Nanosensors are more effective than traditional sensors because of their great sensitivity and selectivity. Metals like platinum, palladium, and gold are used to construct these gas sensors.^[^ ^122^ ^]^ There have been instances when nanoparticles made of gold have been utilized to detect mycotoxin B1 in milk. Sometimes the sensors’ sensitivity is enhanced by having been constructed from single‐walled CNTs and deoxyribonucleic acid (DNA). Liposome nano‐vesicles have been employed for the identification of peanut‐allergenic proteins in pathogens and chocolate. Detection of *E. coli* O157:H7, Salmonella spp., and Listeria monocytogenes utilizing universal protein G‐liposomal nanovesicles and an immunomagnetic bead sandwich test.^[^ ^127^ ^]^ For better agricultural yields, nanosensors are being used to monitor soil conditions. They're useful for finding traces of pesticides on food products, too. Nanosensors have been created to sense not just pesticides but also carcinogens in food items. Time‐temperature integrators and gas detectors are two popular types of sensors utilized in the food packing sector. Nanosensors are utilized in a wide variety of applications, such as array biosensors, nanoparticles in solution, nanoparticle‐based sensors, nano‐test strips, electronic noses, and nano cantilevers. One sort of sensor, “electronic noses,” combine a number of chemical sensors with an information processing system. The sensor is called an electronic nose because it mimics the human nose's performance. There has also been talk of electronic tongue sensors that use the same idea as the electronic nose. When it detects a spoiled ingredient, it changes color to alert the consumer that the meal is no longer safe to eat.^[^ ^122^ ^]^ **Table** **3** clearly represents the nanomaterial‐based biosensors.

**Table 3:** List of nanomaterial‐based biosensors with their application in food science and food nanotechnology

Nanomaterials	Samples	Analyte	References
Titanium dioxide	Food preservation and packaging	Used as whitener in dairy products (e.g., milk and cheese)	[128]
Silicon dioxide	Food preservation and packaging	Act as food colorant, hygroscopic, anticaking, and drying agent.	[129]
Zinc oxide	Food preservation and packaging	Reduces the oxygen flow inside the packed containers	[128]
Inorganic nanoceramic	Food preservation and packaging	Used in cooking (frying)	[130]
Silver nanoparticles	Food preservation and packaging	Acts as antibacterial agent, absorbs and decomposes ethylene in fruit and vegetables	[131]
Polymeric nanoparticles	Food preservation and packaging	Used as bactericidal and efficient delivery mechanism	[132]
Chitosan	Anti‐fungicide	Used as a coating agent for mandarin, strawberries, and fresh fruits	[133]
SWCNT (single‐wall carbon nanotubes)	Wine Honey Phosphate‐buffered solution	Integration with biomolecules Fructose Methyl parathion and chlorpyrifos	[134] [135]
Gold nanoparticles AuNPs	Integration of DNA or enzymes or antibodies with Au NP Pathogens Glucose	Food storage applications Meat and dairy industries Fruit juice	[136]
MWCNT (multi‐walled carbon nanotubes)	Food industry Phosphate‐buffered solution	Integration biomolecules Paraoxon Fructose	[137]
Cu and Au NPs	Surface water	Pathogens	[136]
CdTe QDs (cadmium telluride quantum dot nanoparticles)	Food industry	Integration biomolecules	[138]
ZrO_2_ NPs	Phosphate‐buffered solution	Parathion	[139]
Glyco‐NPs	Phosphate‐buffered solution	*E. coli*	[140]
Exfoliated graphite nanoplatelet xGnPs	Phosphate‐buffered solution	Glucose	[135]
Quantum dots, QDs	Chicken carcass wash water	*Salmonella typhi*	[141]
Silica particles coated with silver shells	Water	*E. coli*	[142]
Gold nanorods	Sodium chloride	*Pseudomonas*	[143]
Au NPs	Milk	*Mycobacterium avium subsp. paratuberculosis*	[144]
(CdSe)ZnS core shell QDs	CH_3_OH/H_2_O (v/v) solvent	Paraoxon (insecticide)	[145]
CdTe QD	Phosphate‐buffered solution	2,4 D (herbicide)	[146]
Au NPs	Glycine buffer	Paraoxon (insecticide)	[147]
CdTe QDs	Phosphate‐buffered solution	Glucose	[145]
Fe_3_O_4_ MNPs	Acetate buffer solution	Glucose	[148]
CdSe@ZnS NPs	Buffer solution	Maltose	[149]
Al_2_O_3_, La, Nano	Oxidation of contaminants	Water purification and soil cleaning	[150]
Silver zeolite	Preservations, disinfectors, and decontaminants	Antimicrobial agent	[151]
Colloidal metals	Enhanced uptake	Food supplements	[152]
Cellulose nanocrystals	Food packaging	Biocompatible high‐water uptake	[153]
Graphene	Detects contaminants in food	Nanoplate‐based nanocomposites	[136]
Magnetic nanoparticles	Pathogen monitoring	Large specific surface area	[154]
Allyl isothiocyanate and carbon nanotubes	Enabled effective storage of shredded cooked chicken meat	Antimicrobial packaging	[155]
Carbon nanotubes	Food inspection and vacuum‐proof food packaging	Optical, electrical, mechanical, and thermal conductivity	[156]
Nanolaminates	Improve the texture properties of foods and serve as carriers	Food‐grade film	[157]

## 5 Natural and Synthetic Nanostructures in Food Industry

Self‐assembled higher‐order structures, like fats and carbohydrates, are among the nano‐sized constituents found in the food system.^[^ ^158^ ^]^ Nanoemulsion, nano‐encapsulate, and food‐grade polymers may all be made using these components, which are distinct from synthetically created nanomaterials/nanostructures.^[^ ^158^ ^]^ The production of nanostructures is not often linked with current nanotechnology, although they are employed in a wide variety of contexts, including the preparation of food, manufacturing, and thermal treatments (e.g., coagulation, homogenizing, or emulsifying). Polysaccharides, proteins, lipids, and other components of food are all spherical particles ranging in size from around 10 to quite a few hundred nanometers. Both emulsification and coagulation depend on the assembly of nanostructures with varying degrees of dimensionality (reticular, 0D, 1D, 2D, and 3D). Corn starch, which is used to produce custard‐like foods, is heated to dissolve its tiny, 3‐D crystalline structures, which are just tens of nanometers thick.^[^ ^159^ ^]^ Milk proteins and casein, two components of milk, are naturally nanostructured. Homogenization of milk results in the formation of fat globules of 100 nm in size.^[^ ^159^ ^]^ The functional and nutraceutical food industries rely heavily on nanotechnology. Colorants and nutritional elements like minerals and vitamins may benefit from NSM encapsulation since it protects them from degradation during processing while also increasing their bioavailability and solubility. Nanoemulsions and nanocapsules (such as micelles and liposomes) are now the most practical NSMs, with certain carbon‐based, green‐synthesized, environmentally friendly nanomaterials also in use. Applications of the nanofood products are described in **Table** **4**.

**Table 4:** Commercial nanofood products and their applications

Product name	Nanomaterial	Type of Product	Manufacturer	Applications	References
Nutra Leaseanola Active Oil	Nanosized self‐assembled liquid structures (NSSL)	Food and beverage	Shemen, Haifa, Israel	Inhibits transportation of cholesterol from the digestive system into the bloodstream	[136]
Fortified Fruit Juice	Micelles 5–100 nm in diameter	Health drink	High Vive. com, USA	Increased Lycopene	[160]
Nanotea	Nanoselenium	Beverage	Shenzhen Become Industry Trading Co. Guangdong, China	Good supplement of selenium	[160]
Nanoceuticals Slim Shake	Conversion of vanilla or chocolate into nanoscale	Health drink	RBC Lifesciences, Irving, USA	Low‐calorie diet	[123]
Tip Top bread	Nanosized self‐assembled liquid structures	Food	George Weston Foods, Enfield, Australia	Nanocapsules of omega‐3 fatty acids	[160]
NanoSlim beverage	Liquid suspended nanoparticle	Food and beverage	NanoSlim	Low‐calorie diet	[123]
Oat Nutritional Drink	–	Food and beverage	Toddler Health, Los Angeles, USA	Contains exactly 33% of all the macro‐ and micronutrients	[161]
Kimchi	Nanometric Lactobacillus plantarum	Korean fermented cabbage dish	Korea	Effective substituent for live probiotics and be useful as a functional ingredient with the anticolitic	[162]
Nano B‐12 Vitamin Spray	Nanodroplets	Food supplements	Nanotech, LLC (USA)	Efficiency enhancement	[136]
Neosino	Silicon	Health supplement	Germany	Health and fitness	[136]
Aquanova	Nanomicelles	Food supplements	Germany	Improve the solubility of vitamins, β‐carotenes, omega fatty acids	[127]
Aquasol preservative	Nanoscale micelle	Food additive	Aquanova	Increases absorption and effectiveness of nutritional additives and preservatives	[127]
Oat Chocolate and Oat Vanilla Nutritional Drink	300 nm of iron particles	Beverage	Oat Chocolate and Oat Vanilla Nutritional Drink	Increases reactivity and bioavailability	[163]
LycoVit	< 200 nm synthetic lycopene	Food additive	BASF	Potent antioxidant and used in soft drinks	[164]
Omega‐3	Nanocochleates as small as 50 nm	Food additive	Bioral	Effective addition of omega‐3 fatty acids	[165]
Nanosilver cutting board	Nanoparticles of silver	Food contact material	A‐Do Global	Potent antibacterial	[164]
Antibacterial kitchenware	Nanoparticles of silver	Food contact material	Nano Care Technology/NCT	Increased antibacterial properties	[164]
Fresher Longer TM Miracle	25 nm of silver nanoparticles	Food storage	Sharper Image, USA	Antimicrobial protection	[160]
Fresher Longer TM	Plastic	Food storage	Sharper Image, USA	Longevity of food products	[160]
Nano Silver Food Containers	Silver	Food storage	A‐DO Global, Korea	Storage	[160]
Food storage containers	Silver	Food storage	BlueMoonGoods, LLC, USA	Food storage	[160]
Nano Silver Baby Milk Bottle	Nanosilver	Health benefits for toddler	Baby Dream Co. Ltd. (South Korea).	Storage	[160]
Nano‐silver Salad Bowl	Silver	Food storage	Changmin Chemicals, Korea	Storage	[166]
Large Kitchen Appliances	Nanosilver	Food storage	Daewoo Refrigerator, Korea	Strong disinfection and storage power	[160]
Nano Storage Box	Silver	Food storage	BlueMoonGood, USA	Food storage	[160]
Novasol	Nanomicelle	Sustain beverage	Aquanova, Germany	Introduce antioxidant into food and beverage products	[160]
Nutri‐NanoTM CoQ‐10	(≈30 nm size)	–	Solgar (USA)	Increased absorption fat	[167]
Nanoceuticals	Nanocolloidal silicate mineral and Hydracel	Nanosized powders	RBC Life Sciences Inc. (USA)	Neutralize free radicals, lower the surface tension of drinking water, and increase solvent properties	[160]
LifePak Nano	–	–	Pharmanex (USA)	Increases bioavailability	[160]
C.L.E.A.N. Products	Nanostructured bioregulators	–	SportMedix, Inc. (USA)	Normal functioning of organs and tissues	[160]
Nanocochleate nutrient	Phosphatidylserine‐based carrier system (≈50 nm) derived from soya bean	–	BioDelivery Sciences International's Bioral	Delivery system for micronutrients and antioxidants	[160]
NanoCluster	Nanoclusters of Artichoke, spirulina, and slim shake chocolate that contain cocoa nanoclusters	Spirulina nanoclusters	RBC Life Sciences Inc. (USA)	Enhances favor	[160]
Lypo‐Spheric Vitamin C	Liposomal nanospheres	Supplements	LivOn Labs, USA	Health application	[160]
Nanocochleate nutrient	Phosphatidylserine‐based carrier system (≈50 nm) derived from soya bean	–	BioDelivery Sciences International's Bioral	Delivery system for micronutrients and antioxidants	[160]
SoluE	–	Vitamin E	Aquanova	Protects stomach from acidic environment	[160]
Daily Vitamin Boost	Silver nanoparticle	Fortified Jambu Juice	Hawaii, USA	Rich in 22 essential vitamins and minerals	[160]
SoluC	‐–	Vitamin E	Aquanova	Protects stomach from acidic environment	[160]
OilFresh	–	Nanoceramic product	US‐based Oilfresh Corporation	Suppresses oil breakdown	[168]
Megace ES	–	Nanocrystal dispersion with micronized particles	Par Pharmaceutical, Inc., Bristol‐ Myers Squibb company, New York, USA	Appetite stimulant in case of cachexia	[160]
Bioral	Calcium ions in GRAS phosphatidylserine from soya bean	Nanocochleate	BioDelivery Sciences International	A protective delivery system for micronutrients and antioxidants against enzymatic degradation	[10]
NanoSil‐10	Silver solution	Supplemented functional drink	Greenwood Consumer Products, USA	Antibacterial	[160]
ASAP Health Max 30 and other silver products	Silver NPs	Supplemented functional drink	American Biotech Labs, USA	Antibacterial	[160]
Silvix3	Silver NPs	Supplemented functional drink	Natural Care Products, USA	Antibacterial and antifungal effects as a surface disinfectant	[160]
MaatShop Crystal Clear Nano Silver	Silver NPs	Supplemented functional drink	MaatShop, USA	Antibacterial	[160]
Nano Colloidal Silver	Silver NPs	Supplemented functional drink	Natural Korea Company Ltd, Korea	Sterilization and quality control	[160]
Nano Silver Sol	Silver NPs	Supplemented functional drink	Phoenix P.D.E. Co Ltd, Korea	Antibacterial activity and sterilization effect	[160]
Sovereign Silver	Silver hydrosols	Supplemented functional drink	Natural‐Immunogenics Corp, USA	Sterilization and quality control	[160]
MesoSilver	Silver NPs	Supplemented functional drink	Purest Colloids, Inc., USA	Highest bioavailability	[160]
Utopia Silver Supplements Advanced Colloidal Silver	Colloidal silver	Supplemented functional drink	Utopia Silver Supplements, USA	Sterilization	[160]
Colloidal Silver Liquid	Silver NPs	Supplemented functional drink	Skybright Natural Health, New Zealand	Supports immune system and defense for natural healing	[160]
Colloidal silver	Colloidal silver consists of small nanoparticles of metallic silver	Food supplement	FairVital, Germany	Colloidal silver particles can be excreted	[160]
Sovereign Silver (8 oz)	Actively charged nanocolloidal silver hydrosol	Food supplement	Natural‐Immunogenics Corp, USA	Safely supports immune system	[160]
Silver (16 oz)	Silver	Food supplement	Activz, USA	Support natural healing.	[160]

## 6 Role of Nanotechnology in Food Functioning

### 6.1 Nanoparticles for Defense from Chemical Corrosion

Deterioration in food quality is caused by several chemical interactions between food's constituent elements and the surrounding environment. Numerous nanomaterials have been found by researchers as effective in preventing these unwelcome reactions in a wide range of food media. Some of these nanomaterials of metal and metal oxides, however, are hazardous due to their ability to produce reactive oxygen species (ROS) and induce oxidative stress, which in turn disrupts the cell's redox equilibrium.^[^ ^169^ ^]^ Therefore, nanomaterials with lower reactivity are used as an antioxidant carriers.^[^ ^170^, ^171^ ^]^ Bioactive substances like vitamins and flavonoids may be encapsulated in polymeric nanoparticles and released in an acidic atmosphere like the stomach.^[^ ^172^ ^]^ Browning, the transformation of phenolics to dark‐colored pigments in aerobic conditions, may be prevented in freshly cut fruits with the use of antioxidants and edible coatings.^[^ ^173^ ^]^ Nano‐zinc oxide has been employed as coated active packing to extend the freshness of sliced Fuji apples, even though only a small number of nanomaterials have been used as antibrowning agents directly.^[^ ^174^ ^]^ The elimination of chemical toxicants or the improvement of nano‐sized nutritional supplements are both ways in which nanotechnology might improve the functional properties of food. Nutraceuticals such as lycopene, b‐carotenes, and phytosterols are integrated into the carriers to reduce the body's cholesterol levels.^[^ ^175^ ^]^ It is well known that food contains a small number of nanostructures that may produce their own nano‐effects. For instance, the efficient absorption of selenium is facilitated by the nano‐selenium concentration of green tea, which has various health advantages. Nano‐encapsulation, or packing material in nanocapsules, is a nanoscale procedure that guarantees the finished product's functioning by allowing for the precise release of core. Hence, encapsulated substances have several benefits, such as the potential to deliver many active chemicals all at once, to last longer on store shelves, to be more stable, and to have their release rate adjusted by factors such as pH.^[^ ^176^, ^177^ ^]^ For more precise, efficient, and useful uses in the food system, Liang et al.^[^ ^177^ ^]^ encapsulated epigallocatechin gallate (EGCG in zein/chitosan) NPs. In comparison to nanoparticles without zein/chitosan (CS) covering in the fatty simulant of 95% ethanol (C_2_H_5_OH), zein/CS NPs were shown to have a greater release of EGCG and DPPH scavenging capabilities. These conclusions corroborated that antioxidant activity and controlled release of EGCG from zein/chitosan nanoparticles in a fatty additive of 95% ethanol shield fatty food products from chemical degradation by enhancing antioxidant performances, which might aid fatty foods in protecting against oxidation for a longer time period.

### 6.2 Nanoparticles for Improving the Physical Properties of Food and Packing Resources

The physical qualities of both packaging and food materials have been proven to be greatly improved by the use of created nanomaterials.^[^ ^178^ ^]^ Many useful properties, including protection from UV radiation^[^ ^179^ ^]^ and high flame resistance,^[^ ^180^ ^]^ were discovered in polymer nanocomposites containing layered silicates in the 1990s. More than a few NPs have been created to improve the outward look of food. The USFDA has given TiO_2_ the green light to be used as a food additive colorant, with condition that its concentration in finished products would never exceed 1% (w/w).^[^ ^181^ ^]^ Color additive mixtures may also include TiO_2_, as well as SiO_2_ and/or Al_2_O_3_, although the USFDA strongly forbids the use of carbon black for this purpose.^[^ ^182^ ^]^ Alternatively, SiO_2_ is utilized as an anti‐caking agent to preserve powdered goods’ flow qualities and also as a carrier of scent in both edible and non‐edible items. According to EU Directive E551, nano‐sized SiO_2_ may legally be used in food items throughout the market.^[^ ^96^ ^]^ β ‐carotene is a nutraceutical constituent that may be used as a coloring agent in addition to functioning as a provitamin A. However, the inclusion of this component into meals is restricted due to its poor level of chemical stability and its hydrophobicity. The nanomaterial that Mehrad et al.^[^ ^183^ ^]^ developed was encapsulated inside solid lipid nanoparticles which incorporated palmitic acid as well as maize oil and then stabilized via whey protein isolate (WPI). This was done so that the physicochemical stability of β‐carotene could be improved. Within this nanostructure, the β‐carotene that had been encapsulated was shielded by a solid shell comprised of crystals of palmitic acid that covered the oil droplets’ surface. This shell safeguarded the β‐carotene. Corn oil was able to reduce the omission of β‐carotene from the solid lipid matrix onto the surface of solid lipid NPs, but WPI was able to enhance the stability of colloidal system, which resulted in an improvement in the oxidative stability of β‐carotene.

## 7 Nanotechnology in Protection of Food

The safety of food is becoming an increasingly important issue for public health across the world. The basic objective of food protection is to guarantee that the food, in both its prepared and consumed states, will not inflict any damage on the individual consuming it.^[^ ^184^ ^]^ During the production, storage, and distribution of the food, it is essential that the food be shielded from any potential for physical, chemical, or biological contaminants.^[^ ^185^ ^]^ Recent advancements in nanotechnology have brought about a revolution in the food industry. This is due to the numerous applications of nanotechnology in processing of food, security and safety of food, along with nanotechnology's strides in improving nutraceutical value, expanding service life, and reducing waste from packaging.^[^ ^185^ ^]^ In today's world, food safety is a serious problem owing to the fast evolution of both culinary traditions and eating patterns. Pathogens, poisons, and other pollutants that are ingested via food may pose significant dangers to human health. The traditional techniques for identifying infections and the poisons they produce require a lot of manual effort and a significant amount of time. Nanotechnology advancements have sped up the process of resolving food safety concerns related to microbiological contamination and have enhanced toxin identification, packaging strategies, and shelf‐life.^[^ ^175^ ^]^ In addition, nanomaterials, such as QDs, carbon nanotubes, metal nanoparticles, and other nanomaterials that are active, may be utilized to construct biosensors for measurement of the microorganisms and other tests that are employed for applications related to food protection.^[^ ^175^, ^185^, ^186^ ^]^

### 7.1 Nanotechnology for the Detection of Foodborne Pathogens

Nano‐biosensors are bioanalytical devices that are generated by utilizing a wide variety of NSMs in conjunction with biological receptors in the construction of an integrated system.^[^ ^187^ ^]^ There have been several different kinds of biosensors created in order to spot food‐borne pathogens and elements that cause food to degrade.^[^ ^188^, ^189^ ^]^ SERS is used as nano‐biosensing method for the purpose of detecting microbial infections in a quick and precise manner.^[^ ^190^, ^191^ ^]^ As a result of their ability to boost Raman signals, Ag nano colloids are often used in Surface‐enhanced Raman scattering^[^ ^192^ ^]^ for the purpose of bacterial detection. Graphene oxide,^[^ ^193^ ^]^ carbon nanotubes,^[^ ^194^ ^]^ magnetic beads,^[^ ^195^ ^]^ silver nanoparticles,^[^ ^58^ ^]^ and plasmonic gold^[^ ^88^ ^]^ are some of the other materials that are routinely employed to identify food‐associated bacterial pathogens. Moreover, nano‐barcodes composed of synthetic genetic material (DNA) molecular beacons that are labeled with colored probes are used in the process of identifying food pathogens.^[^ ^116^ ^]^ The measurement and recognition of light that is dispersed by the cells has made it feasible to directly identify *E. coli* in samples of food. This has made it probable to directly sense *E. coli* in food samples. This kind of sensor works by interacting with a known protein and is described as a bacterium that is grown onto a silicon chip. It is capable of binding with another *E. coli* bacteria that could be found inside the specimen.^[^ ^196^ ^]^ An immunosorbent test based on an array was developed by Chen and Durst^[^ ^197^ ^]^ to detect *E. coli* O157:H7, Salmonella spp., and L. monocytogenes in pure and mixed cultures by employing protein G‐liposomal nano‐vesicles. The researchers proved that protein G‐liposomal nanovesicles are efficient as universal immunoassay reagents and revealed that the protein G‐liposomal nano‐vesicles may be effectively employed in immunoassays for the synchronized recognition of foodborne pathogens. To speed up the detection of *E. coli* O157:H7 in liquid trials, DeCory et al.^[^ ^198^ ^]^ devised an immunomagnetic bead immune‐liposome fluorescence test. Results showed that *E. coli* O157:H7 in aqueous samples could be detected promptly utilizing a combination of immunomagnetic beads and sulforhodamine B encapsulated in immunoliposomes. Others have looked at liposome‐based approaches for detecting pathogens as well.^[^ ^199^, ^200^ ^]^ Pathogens and proteins, which pulsate at varying frequencies according to their biomass, may be recognized and detected using nanosensors like nano‐cantilevers made from silicon‐based materials.^[^ ^201^ ^]^ Over the last several years, many novel nanoparticle‐based detection technologies have been created. To facilitate the targeted binding and visible detection of *Klebsiella, Tominaga* ^[^ ^202^ ^]^ created lateral‐flow immunological trial strips using palladium NPs. Even further, a single *E. coli bacterium* was identified by Thakur et al.^[^ ^203^ ^]^ utilizing a field‐effect transistor system based on reduced graphene nanoparticles. *Cronobacter sakazakii* is bacteria that may be harmful to newborns. Therefore, one of the research groups developed an electrochemical sensing device that is centered on graphene oxide‐gold nanoparticles to identify *Cronobacter sakazakii* in baby formula powder with a limit of detection of 2.0 101 CFU mL^−1^.^[^ ^204^ ^]^ In addition, Song et al.^[^ ^205^ ^]^ created a fluorescence sensing system that employs immunomagnetic NPs paired with liposome NPs. This platform can identify *Cronobacter sp*. at the genus level with a limit of detection of 5.9 103 CFU mL^−1^. In addition, an optical sensing system that is based on aptamers and gold nanoparticles for the purpose of detecting Salmonella in pork trials that were contaminated was created.^[^ ^206^ ^]^ **Table** **5** shows the many kinds of nanoparticles that may be used to detect numerous foodborne pathogens, along with the limit of detection for each type of nanoparticle.

**Table 5:** Nanoparticles employed for the detection of foodborne pathogens

Nanoparticles	Pathogens	Detection limit	Reference
Gold nanorod	*Escherichia coli O157:H7*	1–10 CFU mL^−1^	[223]
Gold nanoparticle	*Salmonella entrica serotype Typhii*	98.9 CFU mL^−1^	[224]
Gold/silicon nanorod	*Salmonella entrica serotype Typhii; Respiratory syncytial virus*	Not reported	[225]
Magnetic bead/quantum dot	*E. coli 0157:H7*	10^3^ CFU mL^−1^	[226]
Quantum dot	*Salmonella entrica serotype Typhii, E. coli O157:H7, Listeria monocytogenes*	103–106 cells mL^−1^	[141, 227, 228, 229]
RuBpy doped silica	*E. coli O157:H7*	1 cell mL^−1^	[226, 230]
Magnetic nanoparticle	*E. coli O157:H7, S. aureus, S. epidermidis*	10^4^ CFU mL^−1^, 8 CFU mL^−1^, 10^3^ CFU mL^−1^	[231]
Single‐walled carbon nanotube	*E. coli*	Not reported	[231]
Immunomagnetic liposome nanoparticle	*Cronobacter sakazakii*	10^3^ CFU mL^−1^	[199]
Liposome nanoparticles	*Salmonella typhimurium*	10^2^ CFU mL^−1^	[69]
Aptamer‐conjugated gold nanoparticles	*Salmonella typhimurium*	10^4^ CFL mL^−1^	[206, 211]

### 7.2 Nanotechnology for Defense from Allergens

The use of nanotechnology as a fundamental instrument for the management and regulation of food allergies has been documented.^[^ ^207^, ^208^ ^]^ Despite these attempts, several nanomaterials have been shown to induce allergic pulmonary inflammation in human beings.^[^ ^209^, ^210^ ^]^ For example, it has been shown that allergen‐specific immune responses of the Th2‐type may be induced in vivo by female BALB/c mice exposed to SiO_2_ nanoparticles.^[^ ^209^ ^]^ At the injection site, the immunotherapy of allergies that included alum (aluminum hydroxide) as an adjuvant exhibited a number of adverse effects, such as puffiness, cutaneous nodules, indurations, inflammation, and granulomas.^[^ ^210^ ^]^ In an effort to bypass this challenge, scientists have seen the possibility of using polymeric NPs^[^ ^211^ ^]^ and agents that target toll‐like receptors (TLR) as alternate adjuvants.^[^ ^212^ ^]^ An example of an adjuvant would be protamine‐based NPs containing TLR‐9 ligand cytosine phosphate guanine (CpG)‐oligodeoxynucleotides (ODNs). *Protamines* are arginine‐rich peptides that have a molecular weight of around 4 kDa and are extracted from the adult testicles of salmon. *Protamines* are detected in the sperm of salmon. *Protamines* are capable of biodegradation and are effectively employed either as an insulin‐additive^[^ ^213^ ^]^ or for reversal of the activity of heparin throughout surgical procedures.^[^ ^214^ ^]^ Furthermore, nanoparticles based on protamine and containing CpG‐ODN inhibit the Th2‐dominated immunological reaction that is caused by an allergen. As a result, these nanoparticles have shown great promise in allergy immunotherapy as a new carrier system.^[^ ^215^ ^]^ In a similar vein, Gamazo et al.^[^ ^216^ ^]^ noted an immunotherapy that used NPs as an allergen distribution system. This enabled the management, lowered the dosages, and decreased allergen vulnerability to the immunoglobulin E that was bound to mast cells and/or basophils. In addition, during the last several years, researchers have been looking at bioinspired NSMs that are proven to have little to nonexistent harmful effects and hence have the potential to be used in the food business.^[^ ^217^ ^]^ Because of its high sensitivity, ease of use, and comparatively low‐cost effectiveness, label‐free biosensing technologies that are based on LSPR (localized surface plasmon resonance) have garnered a lot of interest. However, difficulties have been evidenced when utilizing these techniques for in situ monitoring of real trials because of the unstable nature of colloidal NPs at certain pH levels and salt content. Previously, Lee et al.^[^ ^218^ ^]^ created a cost‐effective and simple optical fiber encased with aptamer‐modified Au nanorods for rapid analysis of ochratoxin A in grape juice extracts (food mycotoxin causing allergy). They then subjected the fiber to localized surface plasmon resonance, that is, LSPR analysis, which illustrated a considerable limit of detection of 12.0 pm. A fluorescence test that is magnetic, nanoparticle‐assisted, and aptamer‐based was described by Zhang et al.^[^ ^219^ ^]^ as a method for detecting allergens in food matrices. As a consequence of this, the linear range was determined to be 0.4–5 g mL^−1^ (R^2^ = 0.996), and the low detection limit was determined to be 77 ng mL^−1^ with effective selectivity. This was accomplished under optimal conditions. Furthermore, Brotons‐Canto et al.^[^ ^220^ ^]^ assessed the beneficial impacts of poly‐(anhydride) NPs (150 nm) as oral carriers for immunotherapy against experimental peanut allergies. A number of further researches indicated the potentially useful uses of nanotechnologies in allergen immunotherapy and vaccinology.^[^ ^221^ ^]^ Nanoparticle‐based formulations will soon be able to be used in allergy immunotherapy, which will allow for the field's unmet requirements to be addressed.^[^ ^222^ ^]^

### 7.3 Nanotechnology for Preventing Heavy Metal Reduction

There is noteworthy danger of dangerous breakouts because of the emission of heavy metals from nanomaterials.^[^ ^171^ ^]^ The longstanding buildup of heavy metals in food items might have harmful consequences due to their release. Nanoparticles made of metals and metal oxides, such as zinc oxide (ZnO),^[^ ^232^ ^]^ silver oxide (AgO),^[^ ^233^ ^]^ and copper oxide (CuO),^[^ ^234^ ^]^ all elevate intracellular reactive oxygen species (ROS)^[^ ^232^ ^]^ and lead to DNA damage and lipid peroxidation. NPs of silica‐modified magnetite that have been coated with a cationic surfactant are used as adsorbents in a microextraction process that can detect very low concentrations of Ni, Cu, Co, Mn, Pb, and Cd in ecological samples. When complexed with 8‐hydroxyquinoline, the silica‐coated nanoparticles produced using cetylpyridinium bromide are able to solubilize metallic ions.^[^ ^235^ ^]^ Magnetite NPs have been shown to be one of the most appealing and affordable substrates for the retrieval of heavy metals^[^ ^236^ ^]^ from a wide variety of sources, and the remediation of pollutants. To eliminate chromium from water, Zhang et al.^[^ ^237^ ^]^ created Fe@Fe_2_O_3_ core/shells, nanowires, and nano‐necklaces. The capability of aminated magnetic iron oxide NPs to work as adsorbents for the elimination of aqueous heavy metal ions, particularly Cu^2+^, Ni^2+^, Pb^2+^, and Zn^2+^, was recently proven by Lin et al.^[^ ^238^ ^]^ Research disclosed that both the initial rate of adsorption and the adsorption capacity of heavy metal ions improved in tandem with the degree of amination. MgO NPs produced using calcination and sol‐gel procedures removed heavy metal ions from polluted water samples and showed promising results against bacterial infections.^[^ ^239^ ^]^ It was hypothesized from these results that nano‐sized MgO particles may be useful in the treatment of wastewater polluted with bacteria and heavy metals owing to their high removal effectiveness, cheap cost, simple manufacture, and eco‐friendly properties. Lingamdinne et al.^[^ ^240^ ^]^ recently showed that stable iron oxide nanoparticles may be manufactured and reused to remove heavy metals. Carbon NPs with high fluorescence characteristics, such as carbon dots (C‐dots) with a particle size of less than 10 nm and carbon nanoparticles (CNPs) of about 10 nm size or larger, have also proved a wide variability of biological properties, including low toxicity and good biocompatibility, over the years. Simpson et al.^[^ ^241^ ^]^ recently produced, characterized, and validated carbon NPs (66 nm) with glycerol for the determination of heavy metal ions, with a limit of detection as low as 0.30 ppm, using a thermal procedure in the existence of H_3_PO_4_.

### 7.4 Nanotechnology for Inhibition of Biofilm Formation

Biofilms are communities of bacteria that attach to various surfaces and secrete an impenetrable polymeric extracellular matrix.^[^ ^242^ ^]^ Biocorrosion, accumulation, and biofouling are only some of the issues brought on by biofilm development (food processing industries), which starts with the adhesion of free‐floating micro‐organisms to a surface through van der Waals forces.^[^ ^243^ ^]^ Safe for human consumption according to the US Food and Drug Administration, glycerol monolaurate (GML) is an antimicrobial agent effective against a wide variety of Gram‐positive bacteria, including Bacillus anthracis.^[^ ^244^ ^]^ Inhibition of biofilm formation by GML in three distinct S. strains has been shown. infection caused by *S. aureus* and methicillin‐resistant *S. aureus*.^[^ ^245^ ^]^ In addition, antimicrobials are used in the nano‐fibers of filter membranes to inhibit biofilm development.^[^ ^237^ ^]^ Additionally, Shahrokh and Emtiazi^[^ ^246^ ^]^ showed that particles of nanosilver in tiny amounts (0.2 ppm) increased bacterial metabolism, recommending an ideal proportion of nanosilver particles for diverse nanomaterials to avoid biofilm formation. The nanoparticle form of nickel oxide has also been suggested as a possible anticancer and antimicrobial agent. NiO‐NPs with a size range of 10–20 nm were produced and tested for their anti‐biofilm action by Saleem et al.,^[^ ^247^ ^]^ who used an eco‐friendly technique by employing *Eucalyptus globulus* leaf extract. Further, a fungal biofilm comprised of *Penicillium chrysogenum, Alternaria alternata, Aspergillus niger*, and *P. pinophilum* was impeded by zinc oxide nanoparticles, as revealed by Gambino et al.^[^ ^248^ ^]^ The biofilm formation of Klebsiella pneumonia was inhibited by gold nanoparticles that had been conjugated with chlorhexidine.^[^ ^249^ ^]^ *Bacillus subtilis* has shown tremendous promise in a wide range of industrial procedures for the creation of goods with high added value. *B. subtilis*'s biofilm‐forming fermentation process is the root cause of many serious problems in industrial settings. Naked IONs and IONs encapsulated with 3‐aminopropyltriethoxy silane (IONs@APTES) were tested for their effects on biofilm formation, bacterial growth, and cell viability in relation to *B. subtilis* by Ranmadugala et al.^[^ ^250^ ^]^ Significant reductions in bacterial biofilm biomass were detected without impacting cell viability, as shown by the findings. These findings indicated that these nanoparticles could be useful in preventing the formation of bacterial biofilm in a variety of settings. In addition, Thuptimdang et al.^[^ ^251^ ^]^ proposed that one way to lessen the effect of silver nanoparticles on biofilms in both manufactured and natural systems is to manipulate the growth circumstances in order to change the biofilm's physical structure. These new results highlight the need for further investigation into the use of nanoparticles in various fields, including medicine. **Table** **6** describes the various applications in food industries.

**Table 6:** Different types of nanoformulations and their applications in food industries

Nanostructured materials	Methods	Nanoparticles	Applications	References
Biopolymers (proteins or polysaccharides)	Microemulsions	Micelles	Produce glycerides in food products	[252]
Low‐density lipoproteins	Microencapsulation	Fish oil	Food additives—mask odor of tuna fish oil	[136]
Liposomes	Encapsulation	Phospholipids	Integrate food antimicrobials for the protection of food products	[253]
Biodegradable biopolymeric NPs	Encapsulation	Polylactic acid	Encapsulate and deliver drugs, vaccines, and proteins	[136]
Liposomes	Nanoencapsulation	Nanoliposomes	Lipid‐based carriers for antioxidants	[254]
Polymer matrices reinforced in the nanofillers	Nanocomposites	Nanoclays, nanooxides, carbon nanotubes, and cellulose microfibrils	Biodegradable packaging	[136]
Food components integrated with droplets	Nanoemulsions	Colloidal dispersions of droplet	Flavored food products. Milk fortified with vitamins, minerals, and antioxidants	[255]
Fine emulsion droplets	Homogenization or micofluidization	Reducing the size of fat globules	High‐pressure homogenizers in producing finer milk emulsions	[136]
PLA NPs	Encapsulation	Curcumin and quercetin	As bio‐stabilizer	[256]
High‐intensity ultrasound waves	Ultrasound emulsification	Oil and water nanoemulsions	To change the characteristics of treated matters	[163]
PLA NPs	Encapsulation	Leaf extract	Developed a greener approach	[256]
Podophyllotoxin and etoposide	Encapsulation	Poly‐d,l‐lactide nanoparticles (PLA NPs)	Anticancer activity	[44]
Stevioside np	Nanoencapsulation	PEG‐PLA nanoparticles	Developed an antidiabetic nutraceutical	[257]
BSA NPs	Encapsulation Nanoformulations	Tea polyphenols, catechin, and epicatechin	Enhance stability and bioavailability	[258]
Canola oil	Nanoemulsions	Vitamin E	Nutritional benefits and oxidative stability	[259]

## 8 Challenges

There are several obstacles to overcome before nanotechnology may be used to build really novel goods and processes in the food industry. The main problem is making safe and efficient delivery systems that people can eat. These systems need sophisticated but cost‐effective processing. To guarantee the safety of meals, the leaching and migration of nanoparticles from packaging materials into food items is a major cause for worry. Some of the NSMs that have been introduced to a system, either directly or indirectly, may become isolated as people start migrating away from the system. At the nanoscale, the materials act quite differently, and our understanding of how to analyze this phenomenon is still rather restricted. The practical use and safety standards of nanotechnology will be greatly aided by a full comprehension of the nanoscale functions and toxicity of nanomaterials. The possible dangers, questions of toxicity, and environmental concerns related to nanoparticles need to be recognized. It is known that nanoparticles are able to pass the biological barrier and penetrate many tissues and organs. Nanoparticles may be synthesized using a variety of chemical processes, each of which has the potential to produce harmful, non‐ecofriendly byproducts that contribute significantly to environmental contamination. Therefore, it is important to examine a comprehensive threat assessment program, regulatory regimes, biosecurity, and public concerns prior to manufacturing, packing, and consuming nano‐based food items in humans. Furthermore, before commercial use and for the manufacture of antibacterial nanoparticles with environmentally benign properties, in vitro and in vivo investigations involving nanoparticle interfaces with living organisms are required.

## 9 Future Scope

Amazing progress has been made in the fields of food science and research owing to the use of nanotechnology. Tracking, tracing, and monitoring may be used to ensure that food quality is maintained with the use of nanotechnology in the detection of contaminants, pathogens, and pesticides. Nanotechnology is not hindered by factors like a lack of skilled laborers, expensive research, or the inability to afford cutting‐edge machinery. Some nanosystems, however, are still in their early stages of development or are being refined into effective nanocomponents. More wide‐ranging research can be carried out in food industry for widespread application. Both opportunities and threats to safety may be evaluated simultaneously. Silver nanoparticles for protecting Asparagus, poultry meat, fresh cut melon by retarding the growth of aerobic psychrotrophics, yeasts, and molds. Zinc oxide nanoparticles for protecting orange juice, and liquid egg albumen by effectively reducing lactobacillus plantarum, salmonella, yeast, and mold counts without changing quality parameters. Titanium oxide for protecting strawberries by reducing browning, early ripening, and decay. Smart packaging, the creation of antigen‐specific biomarkers, and the combination of nanoparticles to make nanocomposite polymeric films are just a few examples of creative concepts that are progressively becoming reality. Further study might be done for future development and industrial uses. Carbon‐neutral biodegradable compounds are nanocomposites. Consequently, they may soon find widespread use as an element of food packaging. Nano silica, therefore, has economic potential as a coating material for surfaces with tunable barrier qualities. In most cases, the presence of the organism that causes food rotting may be confirmed with the use of antigen‐specific indicators. Using nanosensors (fast, less labor‐intensive, and accurate) to detect food pathogens including viruses, bacteria, and mycotoxins would be simple and fast. The AI‐sensors are best paired with a wireless data connection to transfer data between biosensors and platforms powered by smartphones or other intelligent terminals. AI‐biosensors built on wireless technology are more flexible, add additional devices more quickly, and have low costs. Due to expanding food consumption in tandem with the growing global population over the last several decades, AI has embodied contemporary technology in the food business. The capability of the aforementioned intelligent systems to perform a variety of activities, including determining food quality, categorizing foods, providing control mechanisms, and making predictions have increased demand for them in the food sector. The use of AI in the food sector has been expanding for years for a number of reasons, including quality control, categorization and parameter prediction, food sorting, and food safety. Among the popular methods used in the food industry are ANNs, expert systems, adaptive neuro‐fuzzy inference systems (ANFIS), fuzzy logic, and machine learning. Prior to the implementation of AI, research on food has been conducted to increase public understanding of food as well as to enhance the results relating to food properties and food production. The AI approach has several advantages, and the food business has been using it for years with growing frequency till today. Among the Machine Learning (ML) methods that are utilized in food industry include stepwise linear regression (SL‐R), boosted logistic regression (BLR), random forest regression (RF‐R), ordinary least square regression (OLS‐R), support vector regression (SVM‐R), principal component regression (PC‐R), partial least square regression (PLS‐R), and k‐nearest neighbors’ regression (kNN‐R). According to studies, the application of ML has contributed to decision‐making, deciding cost reduction, and business strategy enhancement to better serve user needs. The food sector has used long short‐term memory (LSTM), an artificial recurrent neural network, to detect pH during the fermentation of cheese. On the other hand, NN has been used to predict the ultimate fouling rate in food processing while GA has been used to determine the optimum parameters in food. ML has shown to be useful in anticipating the UK's food insecurity. In addition, ML has shown the ability to forecast the direction of sales in the food market. Additionally, ML was capable of predicting the amount of food waste produced and providing information about the manufacturing process. Similarly, relevant to consumers is the use of nanosensors in film packaging to detect gases produced when food spoils. Producing, retailing, and consuming communities would all profit from the decreased food waste that would be caused by the widespread use of such sensors to detect rotting. Moreover, the sensors are being integrated into packaging materials using carbon nanotubes, primarily for the recognition of bacteria, hazardous chemicals, and food deterioration. Intelligent and smart food packaging technologies allow for extensive study of potential future packaging materials. In a similar vein, comprehensive research insights might be achieved in order to elucidate the potential of nanofibers in the field of food packaging and regarding the interactions of numerous food components while they are being nano‐encapsulated. In the next years, foods generated from nanotechnology have a good chance of expanding the range of possibilities for the formulation and manufacturing of functional meals. It is possible that nanotechnology may eventually come to dominate the whole field of food processing if certain laws and regulations pertaining to nanotechnology are enacted in order to address the many safety concerns related to this technology. Nanotechnology is expected to become the most sophisticated technology with an endless development rate by the year 2050, according to recent projections. This is because of nanotechnology's potential to create solutions that are friendly both on a micro and a macro scale.

## 10 Conclusion

Nanotechnology is an upcoming revolution that promises enormous benefits across all industries, from manufacturing to medical to the food production sector. Nanomaterials prevent the spoilage of food and nanoparticles are increasingly being employed in the food industry. Preservatives, antimicrobial sensors, flavoring agent, wrapping materials, packed food components, and so on are all examples of nanomaterials and nanoscale food additives used to modify nutritional profiles and enhance product longevity, quality, and appeal. The viruses in food can be detected using nanomaterials as biomarkers for maintaining food security and quality. However, there are still challenges that need to be addressed before nanotechnology may be used to build really novel goods and processes in the food industry. The development of safe and efficient delivery system for food products is a great concern because this requires cost‐effective and sophisticated processing. To guarantee the safety of meals, the leaching and migration of nanoparticles from packaging materials into food items is a major cause for worry. At the nanoscale, the materials act quite differently, and analyzing this phenomenon is still rather restricted. A thorough understanding of the toxicity of nanomaterials and their functions at the nanoscale will be very helpful for the practical application of nanotechnology and safety regulations. Furthermore, in vitro and in vivo studies involving the interaction of nanoparticle with living beings is necessary before their commercial application. Moreover, nanomaterials with antibacterial properties need to be investigated for their toxicity against human beings and environment. Nanotechnology‐derived foods have a significant possibility of broadening the number of options for the formulation and manufacture of functional meals. If specific rules and regulations relevant to nanotechnology are adopted in order to solve the various safety issues associated with this technology, it is likely that nanotechnology may eventually come to dominate the whole area of food production. According to current predictions, by 2050, nanotechnology will be the most sophisticated technology with endless development owing to its potential for providing solutions both at macro and micro scales.

## Conflict of Interest

The authors declare no conflict of interest.
